# The c.119-123dup5bp mutation in human γC-crystallin destabilizes the protein and activates the unfolded protein response to cause highly variable cataracts

**DOI:** 10.1038/s41598-025-90977-2

**Published:** 2025-02-24

**Authors:** Mohd Hussain Shah, Venkata Pulla Rao Vendra, Christian Ostrowski, Zhiwei Ma, J. Fielding Hejtmancik

**Affiliations:** Ophthalmic Molecular Genetics Section, Ophthalmic Genetics and Visual Function Branch, National Eye Institute, National Institutes of Health, Bethesda, MD USA

**Keywords:** Lens, Cataracts, Unfolded protein response, Crystalline, Autophagy, Apoptosis, Aggregation, Genotype–phenotype correlation, PERK-dependent pathway, Mutation, Proteins

## Abstract

Ordered cellular architecture and high concentrations of stable crystallins are required for the lens to maintain transparency. Here we investigate the molecular mechanism of cataractogenesis of the CRYGC c.119-123dupGCGGC (p.Cys42AlafsX63) (CRYGC5bpdup) mutation. Lenses were extracted from wild type and transgenic mice carrying the CRYGC5bpdup minigene and RNA was isolated and converted into cDNA. Expression of genes in the unfolded protein response (UPR) pathways was estimated by qRT-PCR and RNA seq and pathway analysis was carried out using the Qiagen IPA website. Postnatal 3 weeks (P3W) Transgenic mice exhibited phenotypic diversity with a dimorphic population of severe and clear lenses. PCA of RNA seq data showed separate clustering of wild-type, clear CRYGC5bpdup, and severe CRYGC5bpdup lenses. Transgenic mice showed differential upregulation in Master regulator Grp78 (Hspa5) and downstream targets in the PERK-dependent UPR pathway including Atf4 and Chop (Ddit3), but not GADD34 (Ppp1r15a). Thus, high levels of CRYGC5bpdup transgene expression in severely affected lenses induces UPRer and UPRmt stress responses primarily through the PERK-dependent and Atf4/Atf5/Ddit3 pathways respectively, inducing autophagy and apoptosis and thence congenital nuclear cataracts. This effect is correlated to CRYGC5bpdup transgene expression, offering insight into cataract pathogenic pathways and recapitulating the variation in cataract severity in humans.

## Introduction

Cataract, or opacification, of the eye lens is the leading cause of blindness worldwide^1^ Cataracts can be subdivided by the age of onset into congenital or infantile (usually diagnosed in the first year of life), childhood (usually diagnosed in the first decade of life), presenile (occurring before approximately 45 years of life) and age-related or senile (occurring after age 45)^2^ Congenital cataracts tend to be caused by severe disruptions to lens development or homeostasis while age related cataracts result from milder insults to multiple genes interacting in concert with environmental stress^2^ Overall, congenital cataracts are responsible for about 3%-12% of childhood blindness^3^ and about 27%-39% of congenital cataracts are inherited. So far over 44 loci and 32 genes have been associated with congenital cataracts^4^ Overall, the most common pathways implicated in genetic congenital cataracts are crystallins (33%), growth factors and receptors (26%), connexins (18%), membrane proteins (11%), chaperones (4%), and intermediate filament proteins (4%)^5^ Thus, mutations in crystallin genes are a major cause of congenital cataracts.

Crystallins are the most abundant proteins in the human eye lens, contributing 90% of the total weight of the eye lens. The ubiquitous crystallins comprise three families. α-Crystallins are small heat shock proteins and have chaperonin properties, while β- and γ-crystallins belong to a superfamily related to bacterial spore coat proteins and spherulin 3A found in slime molds^6^ Of the three families α-, β-, and γ-crystallins contribute 28, 45, and 24% of the total crystallin protein, respectively^7^ Six types of γ-crystallins: γA- (CRYGA), γB- (CRYGB), γC- (CRYGC), γD-(CRYGD), γN- (CRYGN), and γS-crystallin (CRYGS) have been identified in humans. γA-, γB-, γC-, and γD-crystallins are encoded by tandem genes on chromosome 2q33.3 along with three γ-crystallin pseudogenes (CRYGEP, CRYGFP, and CRYGGP), while the γN-crystallin gene is located on chromosome 7q36.1 and the γS-crystallin gene is located on chromosome 3q27.3. Mutations in CRYGD and CRYGC cause 6 and 4% of genetic cataracts, respectively, while CRYGS is responsible for about 1% and CRYGA and CRYGB, which are expressed at lower levels in humans, cause about 0.25% each^5^ While mutations in γ-crystallins can cause a variety of various types of opacities, those in CRYGC and CRYGD cause predominantly nuclear opacities (81 and 73%, respectively), consistent with their high expression levels in the lens nuclear fibers^5^

Cataracts have classically been thought to occur from light scattering through formation of high molecular weight aggregates (HMW) in the lens fiber cell cytoplasm^8,9^ More recently an alternate pathogenic mechanism involving damage to lens cells with resultant degradation and disarray of the lens microarchitecture has been demonstrated^2^ This often involves induction of the unfolded protein response (UPR) with subsequent apoptosis^10^ This mechanism is most often seen with mutations that severely disrupt lens development and homeostasis, especially crystallin mutations that either denature the protein in a fashion that escapes binding by α-crystallin^11,12^ or are expressed at a high enough level that they simply overwhelm the available α-crystallin in the lens cell.

We have previously shown that autosomal dominant zonular cataracts with high intraocular variability are associated with a 5-base insertion in CRYGC and shown that expression of the mutant crystallin disrupts the lens microarchitecture, causing cataracts in transgenic mice^13–15^ In the present report we demonstrate that the molecular pathological mechanism behind cataractogenesis of the 5 base pair duplication in a human γC-crystallin^14^ gene mutation is through activation of the UPR and subsequent induction of autophagy and apoptosis. This involves activation of the PERK (EIF2AK3)) endoplasmic reticulum UPR pathway and the ATF4 mitochondrial UPR pathway, with the age of onset and severity of the cataracts as well as induction of the UPR, autophagy, and apoptosis being dependent on the levels of CRYGC5bpdup mRNA expressed in the mouse lens.

## Results

**Fig. 1:**
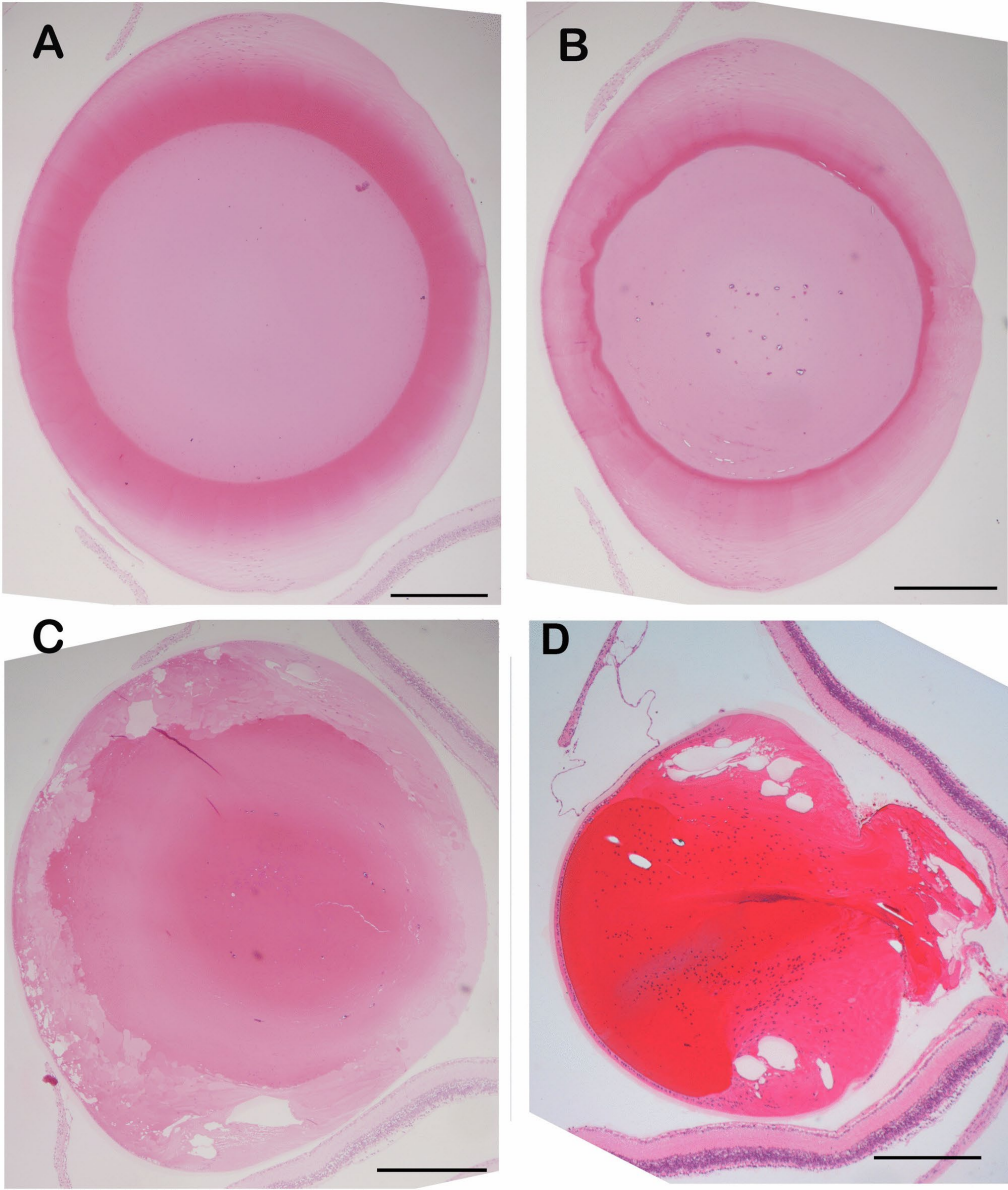
H&E-stained lens sections from 6-week-old mice. (**A**). wild type (**B**). mutant clear (**C**). mutant severe (**D**). 4 week old c.97_357del CRYBA1 (p.Ile33_Ala119del) transgenic mouse lens, shown for comparison.

**Fig. 2:**
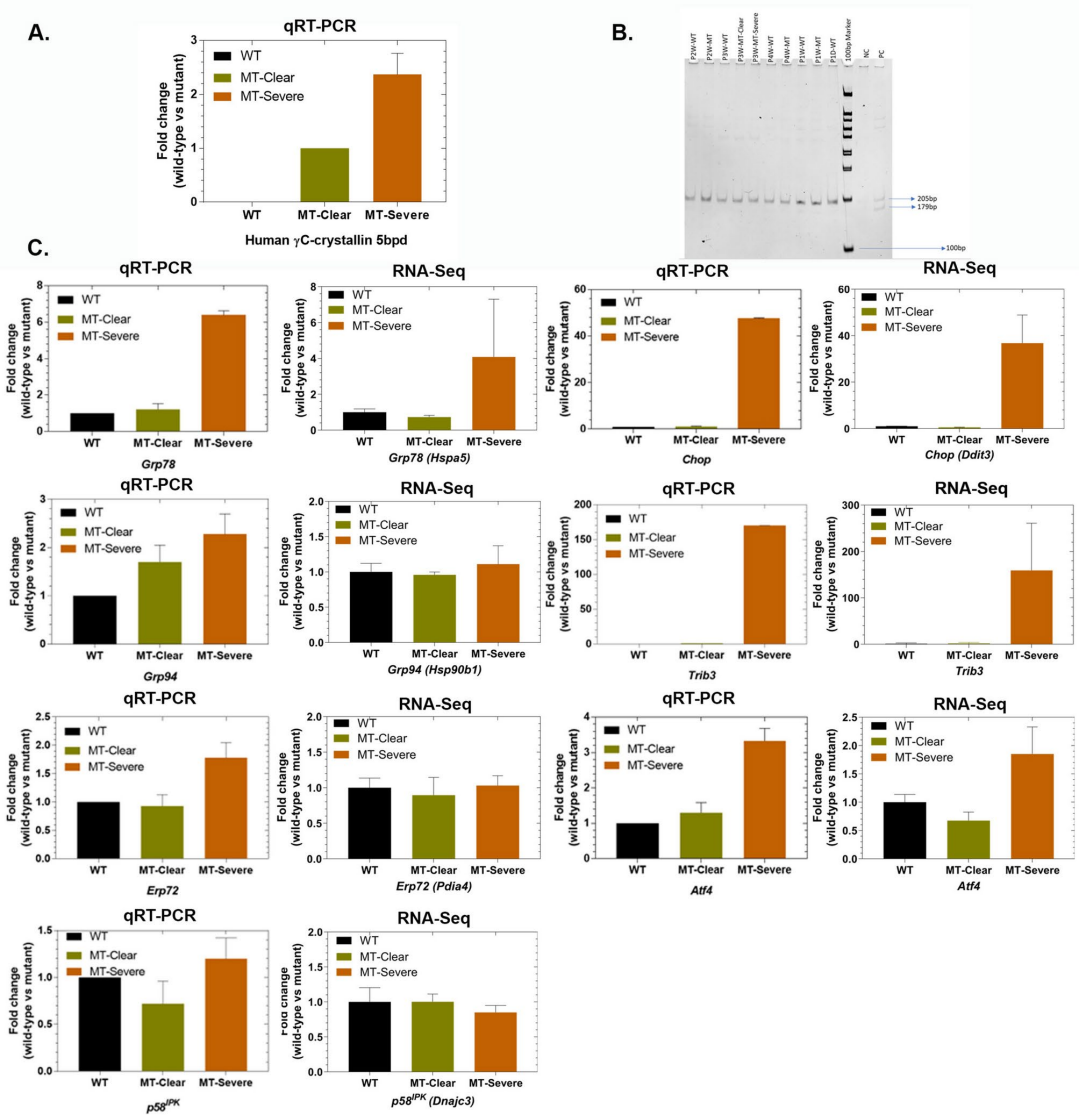
RT-PCR analysis of mRNA levels in wild type (WT) clear mutant (MT-Clear), and severe mutant (MT-Severe) lenses and comparison to RNASeq results. (**A**). qRT-PCR analysis of CRYGC5bpdup transcript. (**B**). RT-PCR analysis of Xbp1: *WT* wild-type lenses, *MT* mutant lenses, *NC* negative control, *PC* positive control (cells treated with tunicamycin, RNA isolated, converted into cDNA and amplified with respective primers covering both the open reading frames of Xbp1). Expression of 5bpdup message in transgenic mice doesn’t alter Xbp1 splicing. C. qRT-PCR analysis of selected mRNAs in UPR/autophagy/apoptosis pathways. For qRT-PCR: wild-type (black) lenses (WT), mutant clear (green) lenses (MT-Clear) and mutant severe (brown) opacification lenses (MT-severe), n = 4.

The transgenic mice having the 5 base pair duplication human γC-crystallin (CRYGC5bpdup) transgene show phenotypic variability in their lenses at 3–6 weeks of age. In some mice both lenses are opaque, in some mice both lenses are clear and in some one lens is clear and the other shows a severe cataract, although even the severely affected lenses are not as badly damaged as lenses of mice transgenic for a c.215 + 1G > A splice mutation in the βA3/A1-crystallin gene (Fig. 1).ref^16,17^ As the cataract severity was essentially dimorphic, we grouped the lenses as ‘wild type’ (WT), ‘mutant severe lenses’ (MT-SEVERE) and ‘mutant clear lenses’ (MC) for the rest of the study. To investigate the cause of the phenotypic variability, we performed a qRT-PCR to quantify the human γC 5-bpd mRNA message and found that on average the mutant severe lenses show over a two-fold increase of 5bpdupCRYGC mRNA compared to the mutant clear lenses (Fig. 2A).The origin of the differential expression is unclear, as it is not inherited in a Mendelian fashion, and both phenotypes can appear in different eyes of the same mouse.

### RNASeq analysis of wild type, clear CRYGC5bpdup, and severe CRYGC5bpdup lenses

**Fig. 3:**
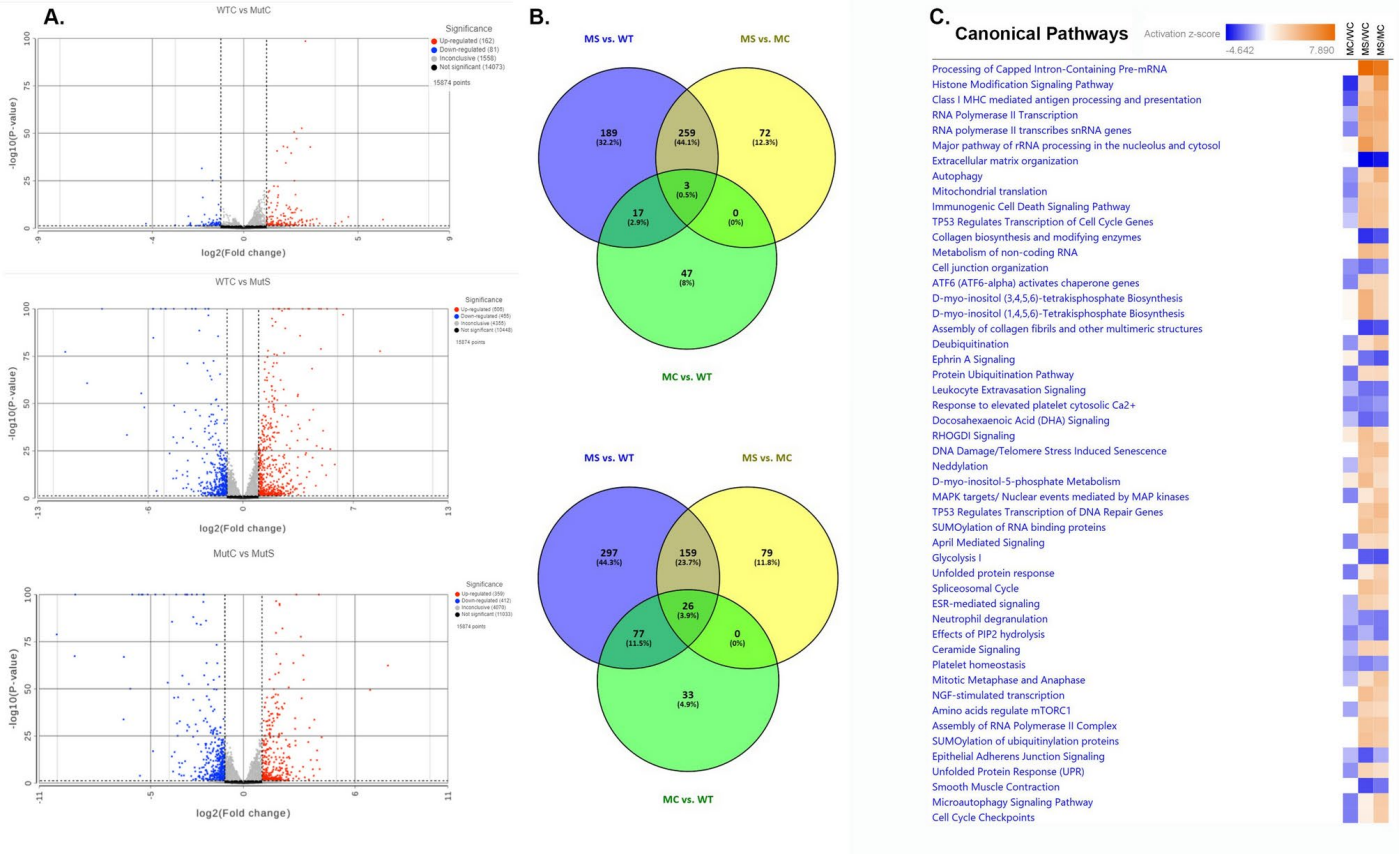
Overview of RNASeq results for wild type clear (WTC), CRYGC5bpdup mutant clear (MutC), and CRYGC5bpdup mutant (MutS) lenses. (**A**). Volcano plot showing differentially expressed genes between WTC, MutC, and MutS lenses (log2Fold Change > 0.4 or <  − 0.4 and adjusted p < 0.05). (**B**). Venn diagram showing two-fold differentially expressed gens among WT, MT-Clear (MC), and MT-Severe (MS) lenses. (**C**). Comparison of the 50 most altered IPA canonical pathways in the WT, MT-Clear (MC), and MT-Severe (MS) mouse lenses and their activation status as reflected in the Z-score for that pathway.

In order to identify the molecular events downstream of CRYGC5bpdup transgene expression RNASeq analysis was carried out on pooled lenses from wild type, clear CRYGC5bpdup, and severe CRYGC5bpdup lenses, even though in some cases clear and severe CRYGC5bpdup lenses came from the same mouse. Transcripts from total of 15,874 genes were identified (Fig. 3A, Table S1), of which expression of 189 genes was increased two-fold between MT-Severe and WT only, 259 genes were increased twofold in both MT-Severe and MT-Clear vs. WT, 72 genes were increased two-fold in MT-Severe vs. MT-Clear only, 47 genes were increased in MT-Clear vs. WT only, 17 genes were increased in MT-Severe and MT-Clear vs. WT but not between MT-Severe and MT-Clear, and 3 genes were increased in MT-Clear vs. WT, MT-Severe vs. WT, and MT-Severe vs. MT-Clear (Fig. 3B). Conversely, expression of 297 genes was decreased two-fold between MT-Severe and WT only, 159 genes were decreased twofold in both MT-Severe and MT-Clear vs. WT, 79 genes were decreased two-fold in MT-Severe vs. MT-Clear only, 33 genes were decreased in MT-Clear vs. WT only, 77 genes were decreased in MT-Severe vs. WT but not between MT-Severe and MT-Clear or WT and MT-Clear, and 26 genes were decreased in MT-Clear vs. WT, MT-Severe vs. WT, and MT-Severe vs. MT-Clear (Fig. 3B). Thus, there was a much greater alteration of gene expression in the severe than clear mutant lenses, both in terms of up- and down-regulation. Expression changes for a subset of UPR constituents including Hspa5, Ddit3, Hsp90b1, Trib3, Pdia4, Atf4, and Dnajc3 were confirmed by qRT-PCR, with the results for Pdia4, hsp90b1, and Dnajc3 showing greater induction than that measured by RNASeq (Fig. 2C).

### Pathway analysis of wild type, clear CRYGC5bpdup, and severe CRYGC5bpdup lenses

The top 50 pathways showing differential expression are shown in Fig. 3C and the full list of altered activity of the IPA canonical pathways is shown in Table S2 with the corresponding Z values. The level of changed expression between WT and MT-Clear lenses is overall much less pronounced than that between WT and MT-Severe lenses, and even between MT-Severe and MT-Clear lenses. In addition, the pathways showing decreased activity in MT-Severe lenses tend to be housekeeping or synthetic in nature, e.g., extracellular matrix organization, collagen synthesis and modifying enzymes, cell junction organization, glycolysis, and epithelial adherens junction signaling. In contrast, pathways showing increased activity in MT-Severe lenses tend to relate to mRNA processing, histone modification, RNA transcription, autophagy, cell death signaling, TP53, MAPK, and ATF6 signaling, ubiquitination and de-ubiquitination, neddylation, sumoylation, ESR-mediated signaling, the UPR, microautophagy, and cell cycle regulation.

**Fig. 4:**
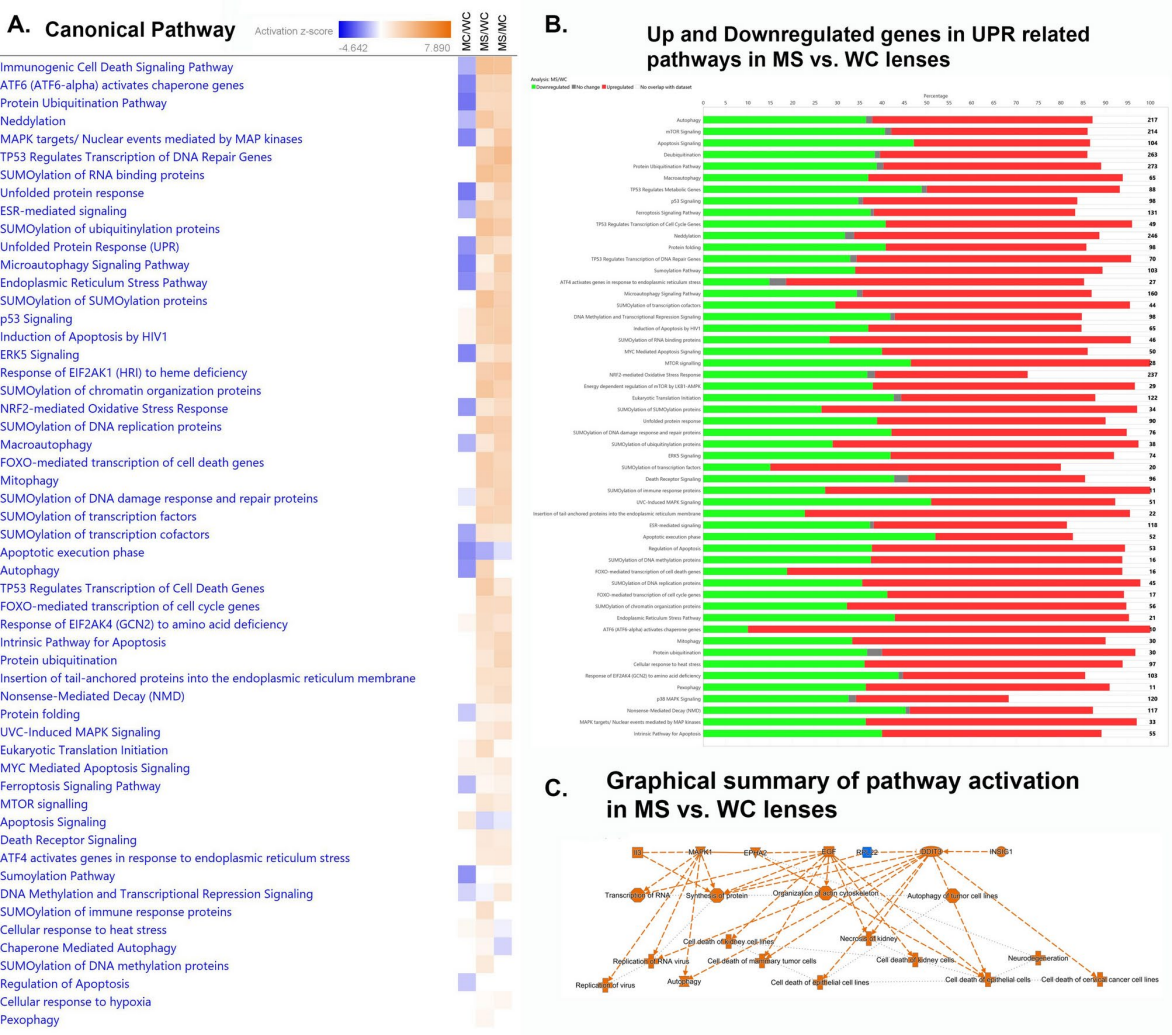
Comparison of UPR related canonical pathways in wild type clear (WC), CRYGC5bpdup mutant clear (MC) and CRYGC5bpdup mutant severe (MS) lenses. Genes upregulated two-fold are analyzed. (**A**). IPA canonical pathways related to the UPR, autophagy, and apoptosis. (**B**). Number of genes in UPR related pathways and the percentage upregulated (red), downregulated (green), unchanged (grey), and not included in the dataset (clear). (**C**). Graphical summary of pathway activation in CRYGC5bpdup mutant severe (MS) compared to wild type clear (WC) lenses. Upstream control molecules are shown at the top of the figure, cellular processes in the middle, and endpoints at the bottom.

Changes in pathways related to the UPR, autophagy, apoptosis and related activities confirms the trend shown by the top 50 pathways and extends activation to include endoplasmic reticulum stress pathways, Eif2ak1 pathways, Nrf2 mediated oxidative stress response, macroautophagy, Foxo mediated cell death pathways, Myc mediated apoptosis signaling, and Atf4 pathways in response to ER stress (Fig. 4A). One interesting anomaly is that apoptosis signaling activity is increased in the MT-Clear vs. WT comparison but decreased in MT-Severe lenses compared to both WT and MT-Clear lenses. Similarly, the apoptosis execution phase activity is decreased in MT-Clear lenses compared to WT and in MT-Severe lenses compared to both WT and MT-Clear lenses, and regulation of apoptosis is decreased in MT-Clear vs. WT lenses and shows little change in MT-Severe lens comparisons. In contrast, other apoptotic pathways such as induction of apoptosis by HIV1, the intrinsic pathway for apoptosis, and MYC mediated apoptosis signaling all show increased activity in MT-Clear vs. WT and in MT-Severe vs. both WT and MT-Clear lenses. An explanation for the mixed activation signals in many of the pathways is seen in Fig. 4B, which shows the percentage and number of genes whose expression is altered in each pathway, and whether their levels are increased or decreased. While a large majority of the genes in some pathways, such as Atf4 activation of genes in response to ER stress, sumoylation of transcription factors, and Atf6 activation of chaperone genes, are activated, others are more evenly balanced, with up to half of the component genes being downregulated. However, the graphical summary of pathway activation in MT-Severe vs. WT lenses shown in Fig. 4C emphasizes that major regulators of the overall lens cell response to CRYGC5bpdup transgene expression include Mapk1, Epha2, Egf, and Ddit3, and the major consequences mostly relate to autophagy and cell death.

### Activation of the endoplasmic reticulum unfolded protein response

**Fig. 5:**
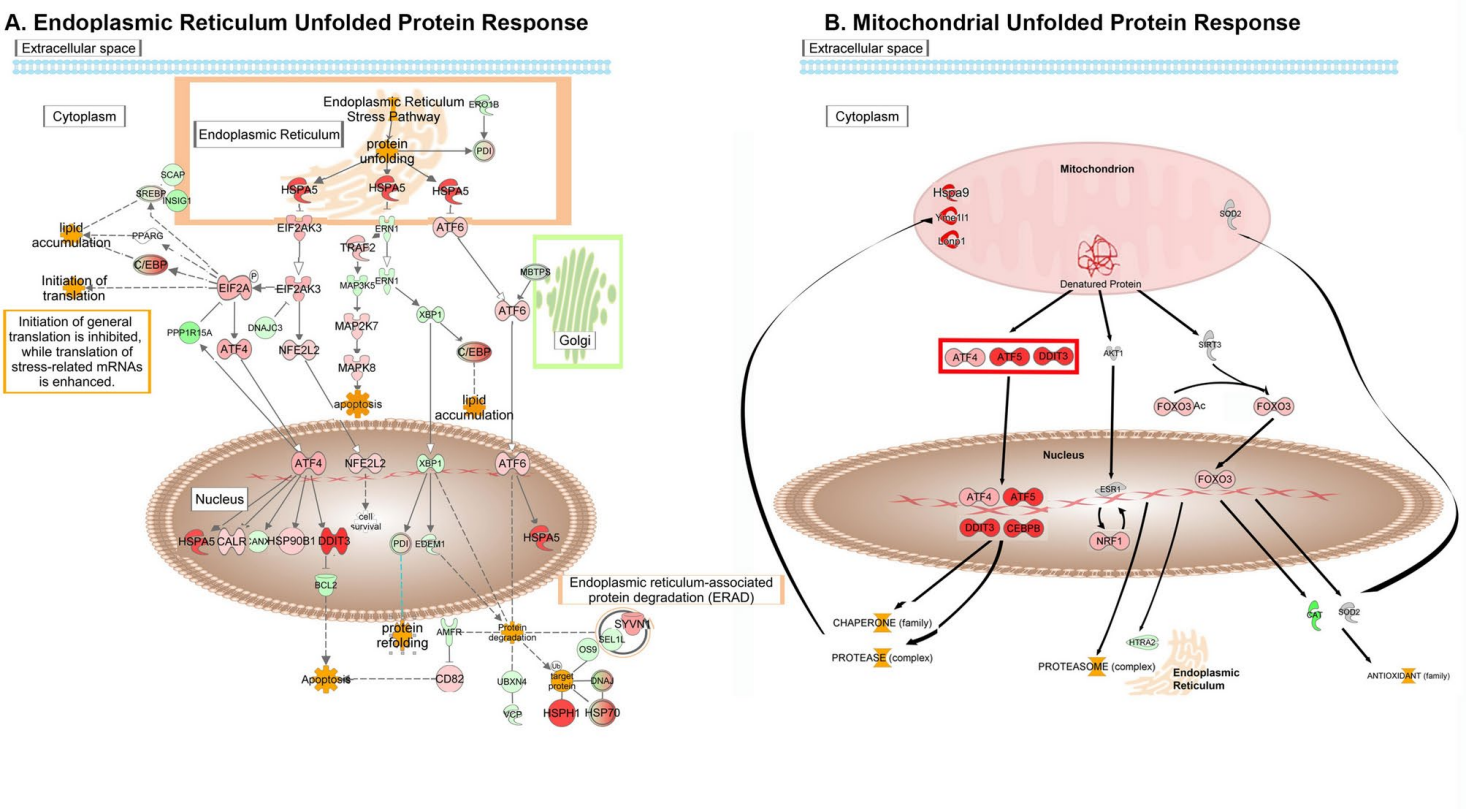
Activation of genes expressed in the UPR. (**A**). Expression changes of genes in the UPRer pathway with the location within the cell indicated. (**B**). Expression changes of genes in the UPRmt pathway. Expression levels are indicated by the shading of genes: increased expression is indicated by red shading, decreased expression is indicated by green shading, and genes whose expression changes less than 1.2-fold are shaded grey. The depth of shading is proportional to the fold change in expression. Processes predicted to be increased are shaded yellow.

In MT-Severe lenses there is an approximately fourfold induction of Hspa5 (Bip, Grp78) associated with increases in Eif2ak3 (Perk) and Atf6, but not Ern1 (Ire1), (Fig. 5A). Eif2ak3 phosphorylates Eif2a, and Eif2a is also increased, as are levels of Atf4 mRNA, resulting in increased transcription of Hspa5 itself, as well as calreticulin, Hsp90b1, and especially Ddit3 (Chop), but not calnexin. Interestingly, both Ppp1r15a (Gadd34) and Dnajc3 (p58) expression are decreased, reducing their negative feedback on Eif2ak3 and Eif2a. As expected with increased Ddit3 (Chop), Bcl2 levels are decreased. Nfe2l2 is also increased by Eif2ak3 (Perk) and activates transcription of genes with antioxidant response elements in their promoters to increase cell survival.

Expression of Ern1 (Ire1), mediator of the second pathway of the UPR^er^ system, is decreased, as is its proximal mediator and endolytic target Xbp1. In addition, there is no increase in Xbp1 cleavage (Fig. 2B), which is required for translation of the functional transcription factor. Despite decreased Ern1 mRNA levels Traf2, which is required for activation of the Mapk8/Jnk and NF-kappaB pathway, is mildly increased, as are Map2k7 and Mapk8 (Jnk), but not the intermediate member of the pathway Map3k5. Overall, activation of this pathway should lead to increased apoptosis.

Atf6, the third mediator of the UPR^er^ pathways, is also increased. The Atf6 protein is cleaved for release from the ER membrane to undergo translation to the nucleus, where it activates the transcription of genes with ER stress response elements (ERSEs) in their promotors, primarily chaperones for ER proteins, but also Hspa5. Separately, synoviolin 1 (Syvn1), part of the ERAD system, is increased as are the holdase chaperonin Hsph1 and Cd82. Conversely its binding partner Sel1l is decreased as are Ubxn4, an integral ER membrane protein and Vcp, which it binds, as is Amfr, all of which should theoretically decrease protein degradation.

### Activation of the mitochondrial unfolded protein response (UPR^mt^)

As the UPR^mt^ can be induced by a variety of conditions that threaten mitochondrial stability including damage to the electron transport chain, accumulation of unfolded proteins, deletion or mutation of mtDNA, inhibition of mitochondrial chaperones or proteases, and increased levels of reactive oxygen species (ROS), it is not surprising that mRNAs encoding various components of this pathway are induced by expression of the CRYGC5bpdup transgene (Fig. 5B). While neither Akt1 nor Sirt3 mRNAs are increased nor is Esr1, their downstream effectors, including Nrf1, which regulates mitochondrial DNA transcription and replication, and Foxo3, which can induce cell death genes to bring about apoptosis as well as antioxidants, show slight increases. However, their targets, including Htra2, catalase, and superoxide dismutase are neutral or decreased. However, the greatest activation of this pathway is seen in the Atf5/Atf4/Ddit3 pathway, which interacts with Cebpb to induce synthesis of chaperones, including Hspd1, hspa9, and hspe1, and proteases including Clpp and Yme1l1 to relieve the stress of unfolded proteins on the mitochondrion.

### Other signaling pathways active in CRYGC5bpdup lenses

**Fig. 6:**
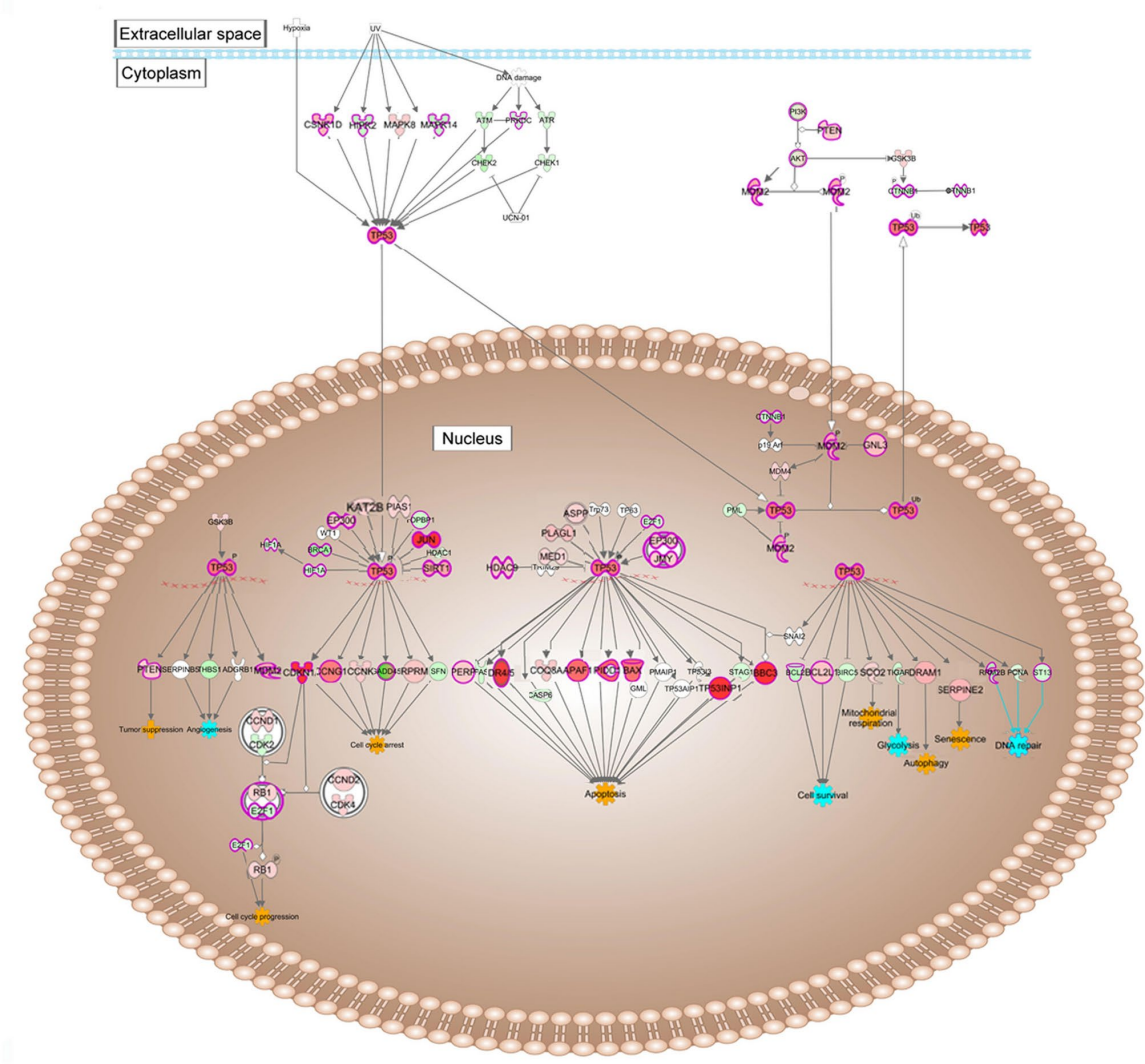
Activation of genes in signaling pathways mediating the UPR influences on autophagy and apoptosis: p53 Signaling pathways. Expression levels are indicated by the shading of genes: increased expression is indicated by red shading, decreased expression is indicated by green shading, and genes whose expression changes less than 1.2-fold are shaded grey. The depth of shading is proportional to the fold change in expression. Processes predicted to be increased are shaded yellow.

The most highly activated control pathways in MT-Severe relative to WT lenses include the signaling pathways of Tp53 and Atf6 (Figs. 5 A and B). Tp53 can be induced by a wide variety of cell stresses, being linked to the UPR^er^ through Mapk8 among others. Tp53 is also linked downstream to cell cycle arrest through Cdkn1a, Ccng1, and Rprm, which are all also increased (Fig. 6). It is linked to apoptosis by Dr4/5 (Tnfrsf10a/b), Pido1, Bax, Apaf1, which activates the caspase cascade, Tp53inp1, which is also linked to autophagy, and Bbc3, which activates apoptosis by permeabilizing the mitochondrial membrane and activate caspases. Finally, it is also linked to autophagy by Dram1. In addition, downregulation of Bcl2 and Birc5, and Tigar decrease cell survival and glycolytic pathways, respectively.

**Fig. 7:**
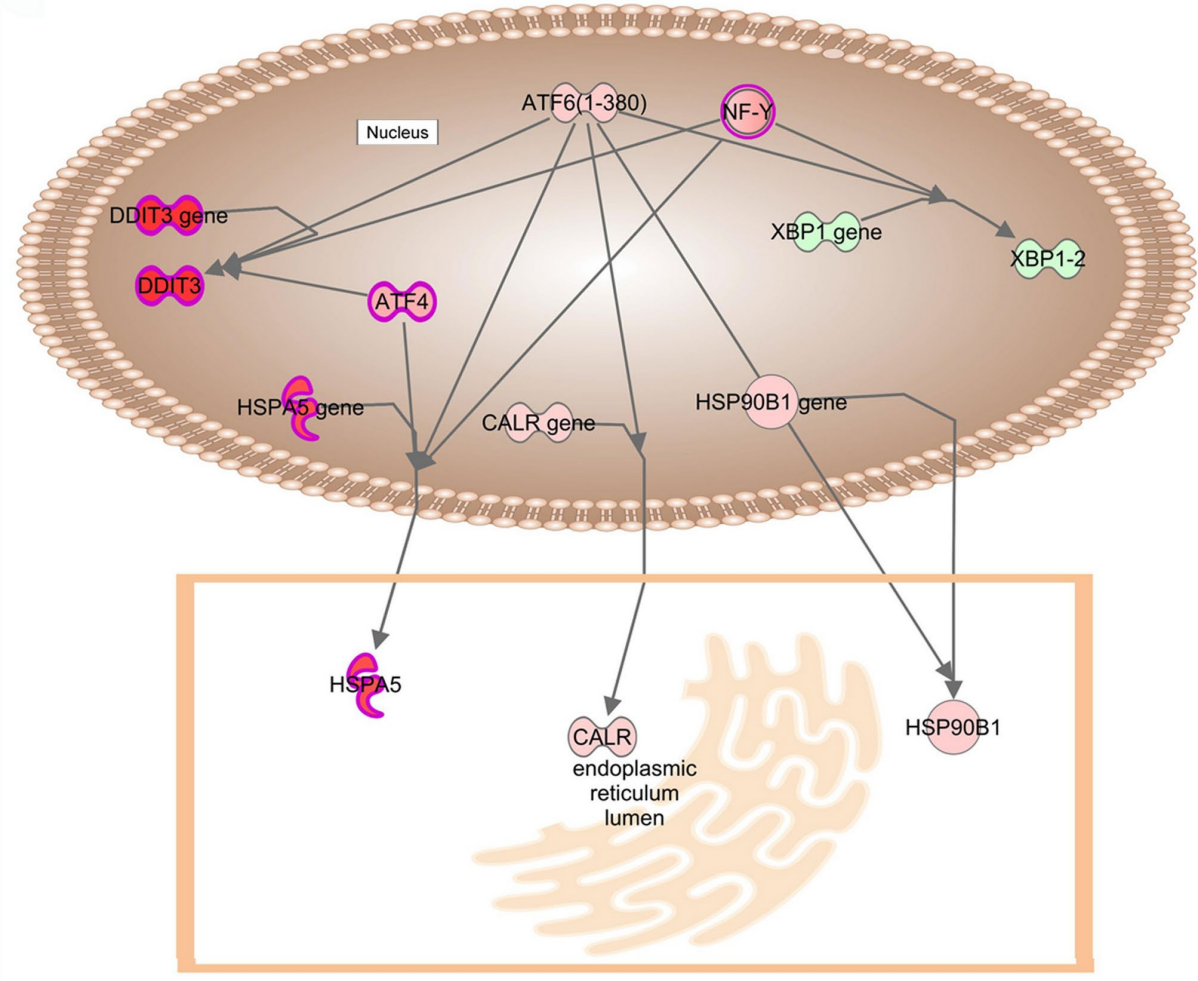
Activation of genes in signaling pathways mediating the UPR influences on autophagy and apoptosis: Atf6 signaling pathways. Expression levels are indicated by the shading of genes: increased expression is indicated by red shading, decreased expression is indicated by green shading, and genes whose expression changes less than 1.2-fold are shaded grey. The depth of shading is proportional to the fold change in expression. Processes predicted to be increased are shaded yellow.

A second control pathway that is highly activated is Atf6 induction of chaperone genes (Fig. 4A, B). In concert with Nf-γ and Atf4, Atf6 induces Ddit3 and also Hspa5, through which it is linked to the UPR^er^ (Fig. 7). It directly induces calreticulin, which plays an important role in protein folding and calcium homeostasis in the endoplasmic reticulum and also directly induces expression of the ER chaperone Hsp90b1.

### Downstream pathways leading to autophagy and apoptosis

**Fig. 8:**
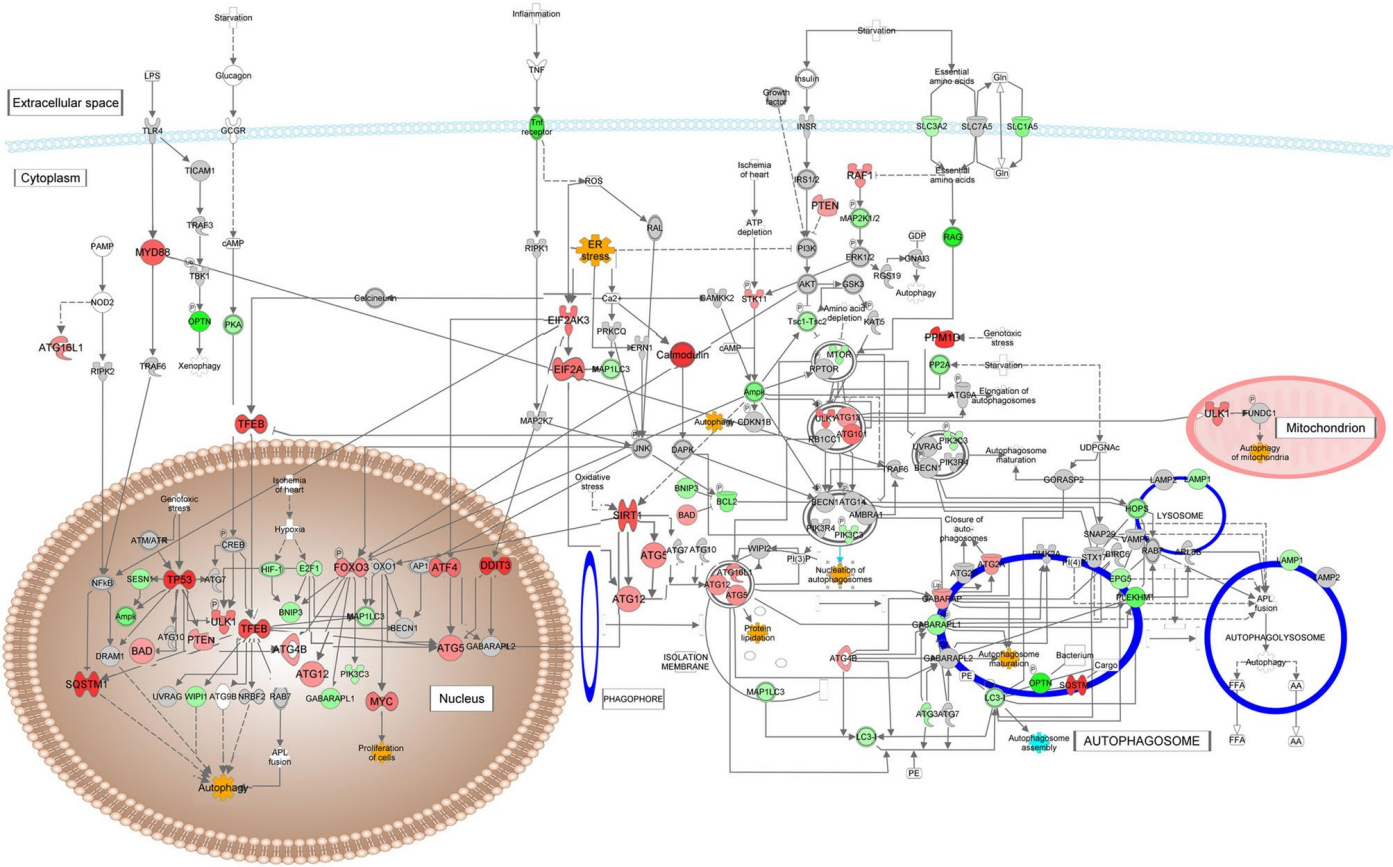
Autophagy and Apoptosis pathways downstream from the UPR: Pathways for autophagy. Expression levels are indicated by the shading of genes: increased expression is indicated by red shading, decreased expression is indicated by green shading, and genes whose expression changes less than 1.2-fold are shaded grey. The depth of shading is proportional to the fold change in expression. Processes predicted to be increased are shaded yellow.

Autophagy pathways are connected to both UPR^er^ and UPR^mt^ through Tp53, Foxo3, Atf4, and Ddit3 (Chop), levels of all of which are elevated in MT-Severe relative to WT lenses (Fig. 8). Tp53 induces Bad, a member of the BCL2 family of regulators of programmed cell death and Pten, a tyrosine phosphatase with a preference for phosphoinositide substrates. Foxo3 elevates levels of a number of autophagy related genes including Atg4b and Atg12, while levels of other downstream mediators of Foxo3 including Gabarapl1 (Atg8), and Pik3c3 are decreased. Atf4 increases ATG5, along with the transcription factor Tfeb, a positive regulator of autophagy that also leads to increased sequestosome, which binds ubiquitin and regulates activation of the Nf-κb signaling pathway. The increased autophagy components lead to increased nucleation of autophagosomes, but mixed effects on proteins involved in assembly and lysosomal fusion, explaining the low Z scores of the autophagosome execution phase pathway seen in Fig. 4A.

**Fig. 9:**
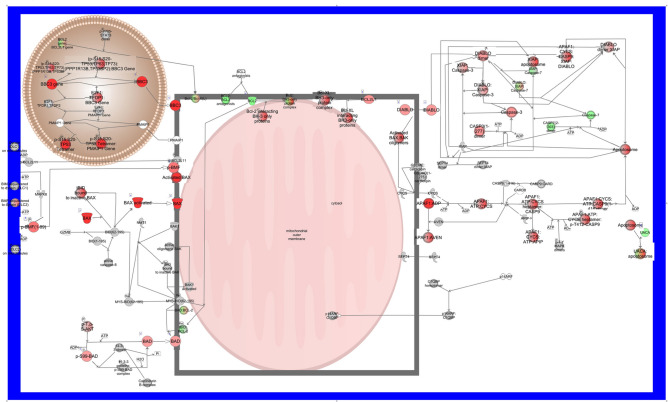
Autophagy and Apoptosis pathways downstream from the UPR: Pathways for apoptosis. Expression levels are indicated by the shading of genes: increased expression is indicated by red shading, decreased expression is indicated by green shading, and genes whose expression changes less than 1.2-fold are shaded grey. The depth of shading is proportional to the fold change in expression. Processes predicted to be increased are shaded yellow.

The main link of the upstream pathways to the apoptosis system is also through Tp53, which increases levels of Bbc3, a Bh3 subclass Bcl2 family member of pro-apoptotic proteins that can cooperate with direct activator proteins to induce mitochondrial outer membrane permeabilization and apoptosis (Fig. 9). Tp53 also induces expression of Bax, another member of the Bcl2 family that forms dimers with Bcl2 to function as an initiator of apoptosis by opening of, the mitochondrial voltage-dependent anion channel (Vdac), leading to loss of mitochondrial membrane potential and release of cytochrome c. Also increased are levels of Diablo, a mitochondrial protein that enters the cytosol when cells undergo apoptosis and allows activation of caspases 3 and 7 by binding to the X-linked inhibitor of apoptosis Xiap, levels of which are decreased in MT-Severe relative to WT lenses. Levels of APAF1 are also increased, and this protein exchanges ADP for ATP and binds cytochrome c to form an apoptosome that can activate caspase 9 and thence contribute to the caspase cascade resulting in apoptosis.

## Discussion

Mice transgenic for a c.119-123dupGCGGC (p.Cys42AlafsX63, CRYGC5bpdup) CRYGC construct driven by the chicken βB1-crystallin promoter (CRYGC5bpdup) develop cataracts with variable age of onset and severity, mimicking the presentation of the CRYGC5bpdup mutation in humans^14,15^ Here, we demonstrate that the severity of the cataracts in CRYGC5bpdup transgenic mice is correlated with expression levels of the transgene. The physical cause of the lens opacity results from disarray and destruction of the lens microarchitecture accompanied by cell death of the lens fiber cells beginning primarily in the bow region of the lens as high levels of βγ-crystallins are synthesized. While the βA3-crystallin promoter does not precisely recapitulate the expression pattern as that of γC-crystallin, they are similarly fiber preferred and should provide a similar pattern for the CRYGC5bpdup mutant mRNA expression^18^.

The histological appearance of MT-Severe lenses is similar to that of lenses from mice transgenic for the c.215 + 1G > A (p.Ile33_Ala119del) splice mutation in the βA3/A1-crystallin gene, but perhaps slightly less severe^17^ Similarly, while both cataract models show activation of the UPR and subsequent apoptotic pathways, this is much more marked in the CRYBA1 splice mutation model, which has essentially all three UPR^er^ pathways including Xbp1 splicing activated, than the CRYGC5bpdup model, in which some pathways appear to be selectively activated relative to others, and some, such as Xbp1 expression and splicing, even appear depressed. In addition, while the normal program of karyolysis during lens fiber development^19^ appears to be intact in the CRYGC5bpdup lenses there appears to be retention of nuclei in the central nuclear fiber cells in the CRYBA1splice lenses, even though a positive TUNEL assay suggests that apoptosis or at least DNA fragmentation, is in progress in these nuclei^17^.

The role of α-crystallins and other chaperones in solubilizing denatured proteins and thus preventing the formation of large aggregates that scatter light is well-described^20–22^ However, only more recently has it been appreciated that cataracts may also be caused by disruption of the lens microarchitecture, which is critically important for transparency, and further, that this disruption is often related to induction of the UPR, increased autophagy of damaged proteins, and if the stress is severe, apoptosis^2,4^ Almost all of the related pathways are elevated in lenses expressing high levels of the CRYGC5bpdup mRNA, with the exception of the apoptotic execution phase, apoptosis signaling, cellular response to heat stress and chaperone mediated autophagy. However, within these broader pathways only some control systems are activated throughout.

The CRYGC and CRYBA1 transgenic models are not the only examples of the UPR and/or apoptosis being involved in cataractogenesis, although some of the examples only identify partial or inconsistent activation of these processes. CRYBA1^17^ CRYGC^15^ and CHMP4B^23^ transgenic mouse cataract models have demonstrated that damage and distortion of lens fiber cells related to the UPR and apoptosis can cause cataracts. In addition, the UPR has been implicated in a number of model systems for cataract, including selenite cataracts in rat lenses^24^ sugar cataracts in galactosemic rat lenses^25^ and transgenic expression of abnormal collagen IV^26^ mutant connexin 50^27^ and αA- and αB-crystallins^28^ in transgenic mice. In human high myopic lens cataracts Yang et al. found that α-crystallins are downregulated and this is associated with increased levels of a number of key elements of the UPR pathways^29^ Interestingly, the same investigators found that different pathways of the UPR are activated in congenital, age-related, and myopic cataracts^30^ Finally, oxidative stress may induce the UPR in lens cells, especially when it overcomes the strong antioxidant defense systems of the lens and the counterbalancing pathways induced by NRF2^31,32^.

In CRYGC5bpdup mice the UPR^er^ Hspa5 (Bip, Grp78) is highly activated, but of the three subsequent pathways, only Eif2ak3 and ATF6 show increased mRNA levels, while Ern1 (Ire1) mRNA levels are actually decreased. While the mRNA levels do not necessarily reflect release of the corresponding protein as Hspa5 binds high levels of unfolded proteins in the ER lumen or phosphorylation of Ern1^33^ the lack of altered splicing of Xbp1 and decrease in Xbp1 mRNA levels strongly suggest that this pathway is not activated. Map2k7 and Mapk8 mRNAs are both mildly increased, but decreased Map3k5 levels suggest that this pathway is only mildly activated, if at all. Conversely, Eif2ak3 (Perk) mRNA is elevated, as are its downstream effectors strongly suggesting that Eif2ak3 is released from Hspa5 and auto-phosphorylates itself and then phosphorylates Eif2a. As Eif2a regulates the initial step of translation it can inhibit general translation to relieve ER stress while enhancing translation of stress-related mRNAs as well as binding to the 5’ untranslated region of Atf4, which translocates to the nucleus to increase transcription of chaperones and UPR target genes including calreticulin, Hsp90b1, Hspa5 (Grp78), and Ddit3 (Chop) (Fig. 5A). In this regard, it should be noted that among the early responding proteins to denatured proteins in the lens are αA-crystallin (Cryaa, Hspb4) and αB-crystallin (Cryab, Hspb5) and other heat shock proteins including Hspb1, although in wild type lenses the latter is expressed at 0.4% and 2.4% of the levels of Cryaa and Cryab, respectively. This is because of the additional role of the crystallins as structural lens proteins requires a constitutive high expression in all sections of the lens^18^ so that their transcriptional regulation is unique to the lens^34^ However in the MUT-Severe lenses expression of all 3 of these chaperones decreases to about 60% of that in WT or MUT-Clear lenses (Fig. S2), an effect also seen in severe acute pancreatitis^35^ chronic allograft nephropathy^36^ and diabetic retinopathy^37^.

After being induced by Atf4, Hspa5 then increases activation of the UPR^er^, while Ddit3 inhibits expression of the antiapoptotic Bcl2, thus inducing apoptosis. Atf6 mRNA is increased in MT-Severe lenses, and after proteolytic processing in the Golgi the activated transcription factor decreases Ern1 and Xbp1 levels as well as Xbp1 splicing, thus inhibiting the UPR^er^, while increasing Hspa5 mRNA levels^38^ Thus, it appears that of the 3 UPR^er^ pathways, the pro-apoptotic Eif2ak3 pathway is highly activated, the relatively anti-apoptotic Atf6 pathway is mildly elevated, and the pro-apoptotic Ern1 pathway is mostly inhibited with some increase in the Mapk8 (Jnk) pathway. Interestingly, Yang et al. found that while mRNA levels of HSPA5, ATF4, spliced XBP1, ATF6 and protein levels of EIF2A, ERN1, and ATF6 were increased in both high myopia and mixed age related cataract lenses, neither protein nor mRNA levels of ATF6 were elevated in mixed congenital cataract lenses, so that the Eif2ak3 pathway is elevated in both human congenital cataracts in the CRYGC5bpdup mouse model^30^ They did not examine elements of the UPR^mt^ or downstream effectors in their study.

Although crystallins exist as soluble cytosolic proteins, approximately 35% of crystallin synthesis occurs on ER bound ribosomes so that the presence of large amounts of unfolded γC-crystallin might be expected to induce the UPR^er^^39^ Elevation of Ddit3 as part of the Eif2ak3 pathway also induces the UPR^mt^ as part of the Atf4 pathway^40^ However, induction of the UPR^mt^ probably also occurs indirectly secondary to cellular stress induced by large amounts of denatured CRYGC5bpdup leading to an oxidative environment in the mitochondria and causing accumulation of misfolded proteins there^41^ As in the UPR^er^, one of the pathways in the UPR^mt^ pathway, the canonical Atf4/Cebpb pathway, is activated so that Atf4, Atf5, and Ddit3 mRNAs are all increased, while Akt1 and Sirt3 mRNA levels are unchanged. Atf4, Atf5, Ddit3, and Cebpb act in the nucleus to increase transcription of a variety of chaperones and proteases that then act in the mitochondrion^42^ In MT-Severe lenses only a single downstream chaperone, Hspa9, but two proteases, Yme1l1 and Lonp1, are increased. Although Nrf1 in the Akt1 pathway and Foxo3 in the Sirt3 pathway are increased, their upstream regulators in the UPRmt show little change, and Akt1 even shows a tendency towards decreasing. This might not fully reflect their functional activities, as Sirt3 is activated in the mitochondria through cleavage by Pmpcb, which would not necessarily be reflected in mRNA levels^43^ Similarly, Foxo3 is primarily activated through deacetylation by Sirt3, which also might not be reflected in its mRNA levels, although these are somewhat increased. However, Foxo3 induces Sod2, which is not elevated in MT-Severe lenses, and catalase, which is significantly decreased, suggesting that this protective arm of the UPR^mt^ is not active or has been overcome. The role of Nrf1 in the UPR is complex, but it is induced by Akt1 acting through Esr1, and the Nrf1 protein is released from the ER and proteolytically processed before translocating to the nucleus, where it is required for induction of most elements of the UPR^mt^ including Atf4, Atf5, Ddit3, and the chaperones Hspa9 and Hspd1^42,44^ Nrf1 also induces Htra2 expression along with other proteasome members in order to clear denatured proteins from the intermembrane space of the mitochondria, but Htra2 levels are also decreased, consistent with progression of the UPR^mt^ from a restorative process towards autophagy and apoptosis.

As mentioned above, the UPR initially stabilizes the ER and mitochondria through increasing production of chaperones and reducing agents, increasing degradation of misfolded proteins through autophagy, and reducing general protein synthesis in favor of stress related proteins. However, under prolonged and severe stress the UPR alters to induce apoptosis. One way in which this occurs is through a change in Eif2ak3 induced alternate splicing of P53 to produce P53/47, an variant lacking the initial 39 amino acids of the full length P53 and producing cell cycle arrest in G2 rather than G1^45^ P53/47 also activates a number of apoptotic target genes by inhibiting both the transcription and translation of Hspa5 and freeing Bik from Hspa5 binding^46^ P53/47 has been reported to induce Bax and Bik along with other apoptotic effectors including Fas, Tnfrsf10b, Birc2, and Tp53ip^47,48^ Of these, Tnfrsf10b and Birc2 are increased significantly in MT-Severe lenses, While none are increased in MT-Clear lenses, suggesting that apoptosis has not fully been induced in this group yet. However, P53/47 P53 is not expressed in the lens, either in the WT, MT-Clear, or MT-Severe mice, although there is a shift in splicing with relatively more increase in the Trp53-202 and 206 isoforms as shown in Fig. S1. These still account for only a minor fraction of the total Trp53 mRNA and the increase in Trp53 activity appears to result from the overall increase in Trp53 mRNA levels.

Another mediator of apoptosis downstream of Tp53 is Bbc3, which is dramatically elevated in MT-Severe lenses, but only slightly in MT-Clear lenses (Fig. 9, Table S1)^49,50^ Bbc3 induces apoptosis through interactions with Bax and Bak, while Bad, which is also increased slightly in MT-Severe but not MT-Clear lenses, acts in a permissive fashion by binding the anti-apoptotic factor Bcl2^51^ In a somewhat similar fashion, IAPs normally bind to caspases and inhibit their activity, thus preventing cell death^52^ Diablo, which is slightly increased in MT-Severe lenses, binds to IAP proteins, allowing Caspase 3 activation and hence apoptosis. In addition, the increased levels of APAF1 in MT-Severe lenses lead to apoptosome formation upon binding with cytochrome c and dATP, and thence activation of Caspase 9 and autophagy^53^.

Overall, cataracts in the CRYGC5bpdup mouse model recapitulate those in human patients in their appearance and intralenticular location, in their variable severity among family members with the identical mutation and even between eyes in the same individual, and in the time course of cataractogenesis, which can include an apparent latent period with clear lenses followed by a relatively rapid progression to a total cataract. These similarities suggest that the pathological mechanism of the cataracts in mice might also be similar to those in humans with the corresponding mutation. The strong correlation between levels of CRYGC5bpdup mRNA and the lens phenotype suggests that this might be the main driver of the cataractogenesis. That differing lens phenotypes can occur in the two lenses of the same mouse argues against, but does not absolutely exclude, positional insertion effects or modifying genes as a cause. Although we currently do not have a good theory to explain the variability in CRYG5bpdup mRNA levels or phenotype, some sort of stochastic process during lens development seems likely based on the available data.

While cataracts in the CRYGC5bpdup mouse model are not the first described as occurring through the UPR, autophagy, and apoptotic pathways, they are currently among the best described models with individual pathways and pathogenic mechanisms delineated in detail. The broad mechanism that this model suggests includes induction of the UPR^er^ by large quantities of misfolded 5bpdup γC-crystallin and the UPR^mt^, perhaps secondarily, with initial induction of chaperones and proteases, most obvious in the MT-Clear lenses, followed in the MT-Severe lenses by a shift towards apoptotic pathways as the lens cell environment deteriorates in the face of the initial UPR response. This model supports and expands evidence for the UPR in congenital cataractogenesis and clarifies the specific sub-pathways and their roles in this process.

## Material and methods

### Generation of transgenic mice

All procedures with the mice were in accordance with the National Institutes of Health guideline on the care and use of animal in research as approved by the National Eye Institute Animal Care and Use Committee, ASP Number: NEI-576) and the ARVO statement for the use of animals in ophthalmic and vision research as well as the ARRIVE essential 10 guidlines. Transgenic mice were generated as described earlier^15^ Briefly constructs were made in PCBB1, which consists of the first 468 bp of the chicken βB1-crystalllin promoter followed by the c.119-123dupGCGGC CRYGC cDNA or wild type (WT) CRYGC DNA and then the final human βA3/A1-crystallin gene splice site, which includes the last 1055 bp of exon5, intron5, and exon6 including the polyadenylation signal. Target fragments were generated by *PmeI* digestion, purified, and injected to pronuclear stage inbred FVB/N embryos^54^.

### Lens extraction

Mice under the age of 7 days with an estimated weight of 5 g (Jackson Laboratory, Body Weight Information for B6 (000664), https://www.jax.org/jax-mice-and-services/strain-data-sheet-pages/body-weight-chart-000664# , accessed 9/11/2024) were sacrificed by cervical dislocation and older mice were sacrificed by carbon dioxide euthanasia. Then, w hile the eye globe was held with forceps, a small incision was made at irido-corneal region with a fine forceps and the lens was extracted from the through the incision, placed on dry ice and stored at -80 °C. A piece of tail was also collected for genotyping and DNA was extracted using DNA isolation kit (DNeasy Blood & Tissue Kit; Qiagen, Valencia, CA). PCR based genotyping was carried out using a set of primers flanking the βB1-crystallin promoter and transgene specific region (Table S3).

### RNA isolation, cDNA synthesis and qRT-PCR

Total RNA was isolated using an RNA isolation kit (The RNeasy Plus Mini Kit; Qiagen, Valencia, CA) and quantified using a spectrophotometer (Nanodrop 2000C; ThermoFisher). A first strand cDNA was synthesized from approximately 0.5 μg of total RNA by cDNA synthesis kit (Super III first strand synthesis for RT PCR kit; Invitrogen) according to the manufacturers protocol. qRT-PCR was performed using Applied Biosystems ViiA7 Real Time PCR system with the following amplification conditions: an initial incubation of the samples at 50 °C for 2 min and denaturation at 95 °C 15 min followed by 40 cycles of denaturation, annealing and extension at 95 °C 15 s, 60 °C 30 s and 72 °C 30 s. *Gapdh* was used as an endogenous control for normalizing the target mRNA. Relative expression of each target gene was calculated using 2^(∆∆Ct) method. The primers were standardized, and efficiencies were tested before performing qRT-PCR. Primers used for the genotyping PCR and qRT-PCR are listed in Table S3.

### RNA-Seq

About 200 ng of RNA samples were used for RNA seq. analysis. Total RNA samples were purified from the lenses of 3-week-old wild-type and KO mice (n = 3) using a Qiagen kit. After passing quality control criteria, RNA-sequencing was carried out using an Illumina NovaSeq platform utilizing a paired-end 150 bp sequencing strategy (Novogene Corporation Inc., Sacramento, CA, USA). Analysis was performed using Partek Flow (https://partekflow.cit.nih.gov/flow) on the NIH Biowulf supercomputing cluster.

### IPA analysis

The networks and, functional analyses were generated through the use of QIAGEN Ingenuity Pathway Analysis (https://digitalinsights.qiagen.com/products-overview/discovery-insights-portfolio/analysis-and-visualization/qiagen-ipa/ )^55^ accessed on 8/19/2024. A data set containing gene identifiers and rpkm values was uploaded into the application. Each identifier was mapped to its corresponding entity in QIAGEN's Knowledge Base. For pathway identification an expression ratio cutoff of 2 × was set to indicate molecules whose expression was significantly perturbed. These molecules, called Network Eligible molecules, were overlaid onto a global molecular network developed from information contained in the QIAGEN Knowledge Base. Networks of Network Eligible Molecules were then algorithmically generated based on their connectivity. Canonical pathways analysis identified the pathways from the QIAGEN Ingenuity Pathway Analysis library of canonical pathways that were most significant to the data set. Molecules from the data set that met the expression ratio cutoff of 2 × and were associated with a canonical pathway in the QIAGEN Knowledge Base were considered for the analysis. The significance of the association between the data set and the canonical pathway was measured in two ways: 1) A ratio of the number of molecules from the data set that map to the pathway divided by the total number of molecules that map to the canonical pathway is displayed; and 2) A right-tailed Fisher’s Exact Test was used to calculate a p-value determining the probability that the association between the genes in the dataset and the canonical pathway is explained by chance alone. 3) In many cases a z-score was calculated to indicate the likelihood of activation or inhibition of that pathway.

Networks are shown as a graphical representation of the molecular relationships between molecules and were prepared using the IPA MyPathways function. Molecules are represented as nodes, and the biological relationship between two nodes is represented as an edge (line). All edges are supported by at least one reference from the literature, from a textbook, or from canonical information stored in the QIAGEN Knowledge Base. Human, mouse, and rat orthologs of a gene are stored as separate objects in the QIAGEN Knowledge Base but are represented as a single node in the network. The intensity of the node color indicates the degree of up-(red) or down-(green) regulation. In order to more fully display the network pathways genes whose expression increased or decreased 1.2 × or greater are shown as increased or decreased. Nodes are displayed using various shapes that represent the functional class of the gene product. Edges are displayed with various labels that describe the nature of the relationship between the nodes (e.g., P for phosphorylation, T for transcription). The UPR^mt^ network is not a canonical IPA pathway but was constructed in the IPA MyPathways function from previously published analyses and shaded using the IPA overlay function^42,56,57^.

## Supplementary Information

Supplementary Information.

## Data Availability

Original data for this report including RNASeq and qRT-PCR results are available at DRYAD: 10.5061/dryad.rn8pk0pmm.

## References

[1] GBD_2019_Blindness_and_Vision_Impairment_Collaborators;_Vision_Loss_Expert_Group_of_the_Global_Burden_of_Disease_Study. Causes of blindness and vision impairment in 2020 and trends over 30 years, and prevalence of avoidable blindness in relation to VISION 2020: the Right to Sight: an analysis for the Global Burden of Disease Study. *Lancet. Global Health* **9**, e144–e160. 10.1016/S2214-109X(20)30489-7 (2021). 33275949 10.1016/S2214-109X(20)30489-7 PMC7820391

[2] Shiels, A. & Hejtmancik, J. F. Inherited cataracts: Genetic mechanisms and pathways new and old. *Exp. Eye Res.* **209**, 108662. 10.1016/j.exer.2021.108662 (2021). 34126080 10.1016/j.exer.2021.108662 PMC8595562

[3] Haargaard, B., Wohlfahrt, J., Fledelius, H. C., Rosenberg, T. & Melbye, M. A nationwide Danish study of 1027 cases of congenital/infantile cataracts: etiological and clinical classifications. *Ophthalmology* **111**, 2292–2298 (2004). 15582089 10.1016/j.ophtha.2004.06.024

[4] Shiels, A. & Hejtmancik, J. F. Mutations and mechanisms in congenital and age-related cataracts. *Exp. Eye Res.* **156**, 95–102. 10.1016/j.exer.2016.06.011 (2017). 27334249 10.1016/j.exer.2016.06.011 PMC5538314

[5] Shoshany, N., Hejtmancik, J. F., Shiels, A. & Datiles, M. Congenital and Hereditary Cataracts: Epidemiology and Genetics. In *Pediatric Cataract Surgery and IOL Implantation)* (ed. Kraus, C. L.) (Springer, 2020).

[6] Mörner, C. T. Untersuchungender protein-substanzen den lichtbrechenden Medien des Auges. *Hoppe-Seyler’s Z Physiol. Chem.* **18**, 61–106 (1894).

[7] Lampi, K. J. et al. Sequence analysis of betaA3, betaB3, and betaA4 crystallins completes the identification of the major proteins in young human lens. *J. Biol. Chem.* **272**, 2268–2275 (1997). 8999933 10.1074/jbc.272.4.2268

[8] Datiles, M. B. 3rd. et al. Longitudinal study of age-related cataract using dynamic light scattering: loss of alpha-crystallin leads to nuclear cataract development. *Ophthalmology* **123**, 248–254. 10.1016/j.ophtha.2015.10.007 (2016). 26545319 10.1016/j.ophtha.2015.10.007 PMC4724511

[9] Benedek, G. B., Chylack, L. T., Libondi, T., Magnante, P. & Pennett, M. Quantitative detection of the molecular changes associated with early cararactogenesis in the living human lens using quasielastic light scattering. *Current Eye Res.* **6**, 1421–1432 (1987). 10.3109/02713688709044506 3427992

[10] Zhou, Y., Bennett, T. M. & Shiels, A. Lens ER-stress response during cataract development in Mip-mutant mice. *Biochim. Biophys. Acta.* **1433–1442**, 2016. 10.1016/j.bbadis.2016.05.003 (1862). 10.1016/j.bbadis.2016.05.003 PMC4885755 27155571

[11] Moreau, K. L. & King, J. A. Cataract-causing defect of a mutant gamma-crystallin proceeds through an aggregation pathway which bypasses recognition by the alpha-crystallin chaperone. *PLoS One* **7**, e37256. 10.1371/journal.pone.0037256 (2012). 22655036 10.1371/journal.pone.0037256 PMC3360035

[12] Rajaraman, K., Raman, B., Ramakrishna, T. & Rao, C. M. The chaperone-like alpha-crystallin forms a complex only with the aggregation-prone molten globule state of alpha-lactalbumin. *Biochem. Biophys. Res. Commun.* **249**, 917–921. 10.1006/bbrc.1998.9242 (1998). 9731236 10.1006/bbrc.1998.9242

[13] Scott, M. H. et al. Autosomal dominant congenital cataract: Interocular phenotypic heterogeneity. *Ophthalmology* **101**, 866–871 (1994). 8190472 10.1016/s0161-6420(94)31246-2

[14] Ren, Z. et al. A 5-base insertion in the γC-crystallin gene is associated with autosomal dominant variable zonular pulverulent cataract. *Hum. Genet.* **106**, 531–537 (2000). 10914683 10.1007/s004390000289

[15] Ma, Z. et al. Overexpression of human γC-crystallin 5bp duplication Disrupts Lens Morphology in Transgenic Mice. *Invest. Ophthalmol. Vis. Sci.* **52**, 5269–5375 (2011). 10.1167/iovs.11-7168 PMC3176079 21436266

[16] Kannabiran, C. et al. Autosomal dominant zonular cataract with sutural opacities is associated with a splice site mutation in the betaA3/A1-crystallin gene. *Mol. Vis.* **4**, 21–26 (1998). 9788845

[17] Ma, Z. et al. Human betaA3/A1-crystallin splicing mutation causes cataracts by activating the unfolded protein response and inducing apoptosis in differentiating lens fiber cells. *Biochim. Biophys. Acta.* **1214–1227**, 2016. 10.1016/j.bbadis.2016.02.003 (1862). 10.1016/j.bbadis.2016.02.003 PMC4870603 26851658

[18] Ma, Z. et al. Patterns of crystallin gene expression in differentiation state specific regions of the embryonic chicken lens. *Invest. Ophthalmol. Vis. Sci.* **63**, 8. 10.1167/iovs.63.4.8 (2022). 35412582 10.1167/iovs.63.4.8 PMC9012887

[19] Brennan, L. et al. Autophagy requirements for eye lens differentiation and transparency. *Cells*10.3390/cells12030475 (2023). 36766820 10.3390/cells12030475 PMC9914699

[20] Horwitz, J. The function of alpha-crystallin: Proctor lecture. *Invest. Ophthalmol. Vis. Sci.* **34**, 10–22 (1993). 8425816

[21] Delaye, M. & Tardieu, A. Short-range order of crystallin proteins accounts for eye lens transparency. *Nature* **302**, 415–417 (1983). 6835373 10.1038/302415a0

[22] Benedek, G. B. Theory of transparency of the eye. *Appl. Optics* **10**, 459–473 (1971). 10.1364/AO.10.000459 20094474

[23] Shiels, A., Mackay, D., Bassnett, S., Al Ghoul, K. & Kuszak, J. Disruption of lens fiber cell architecture in mice expressing a chimeric AQP0-LTR protein. *FASEB J.* **14**, 2207–2212 (2000). 11053241 10.1096/fj.99-1071com

[24] Palsamy, P., Bidasee, K. R. & Shinohara, T. Selenite cataracts: activation of endoplasmic reticulum stress and loss of Nrf2/Keap1-dependent stress protection. *Biochim. Biophys. Acta.* **1794–1805**, 2014. 10.1016/j.bbadis.2014.06.028 (1842). 10.1016/j.bbadis.2014.06.028 PMC4293018 24997453

[25] Mulhern, M. L. et al. The unfolded protein response in lens epithelial cells from galactosemic rat lenses. *Invest Ophthalmol. Vis. Sci.* **47**, 3951–3959. 10.1167/iovs.06-0193 (2006). 16936110 10.1167/iovs.06-0193

[26] Firtina, Z. et al. Abnormal expression of collagen IV in lens activates unfolded protein response resulting in cataract. *J. Biol. Chem.* **284**, 35872–35884. 10.1074/jbc.M109.060384 (2009). 19858219 10.1074/jbc.M109.060384 PMC2791016

[27] Alapure, B. V., Stull, J. K., Firtina, Z. & Duncan, M. K. The unfolded protein response is activated in connexin 50 mutant mouse lenses. *Exp. Eye Res.* **102**, 28–37. 10.1016/j.exer.2012.06.004 (2012). 22713599 10.1016/j.exer.2012.06.004 PMC3461258

[28] Andley, U. P. & Goldman, J. W. Autophagy and UPR in alpha-crystallin mutant knock-in mouse models of hereditary cataracts. *Biochim. Biophys. Acta.* **234–239**, 2015. 10.1016/j.bbagen.2015.06.001 (1860). 10.1016/j.bbagen.2015.06.001 PMC4673011 26071686

[29] Yang, J. et al. UPR activation and the down-regulation of alpha-crystallin in human high myopia-related cataract lens epithelium. *PLoS One* **10**, e0137582. 10.1371/journal.pone.0137582 (2015). 26351848 10.1371/journal.pone.0137582 PMC4564188

[30] Yang, J. et al. Differences in unfolded protein response pathway activation in the lenses of three types of cataracts. *PLoS One* **10**, e0130705. 10.1371/journal.pone.0130705 (2015). 26091066 10.1371/journal.pone.0130705 PMC4475046

[31] Zhang, X. et al. Antioxidant system and endoplasmic reticulum stress in cataracts. *Cell Mol. Neurobiol.* **43**, 4041–4058. 10.1007/s10571-023-01427-4 (2023). 37874455 10.1007/s10571-023-01427-4 PMC10842247

[32] Periyasamy, P. & Shinohara, T. Age-related cataracts: Role of unfolded protein response, Ca(2+) mobilization, epigenetic DNA modifications, and loss of Nrf2/Keap1 dependent cytoprotection. *Prog. Retin. Eye Res.* **60**, 1–19. 10.1016/j.preteyeres.2017.08.003 (2017). 28864287 10.1016/j.preteyeres.2017.08.003 PMC5600869

[33] Shreya, S., Grosset, C. F. & Jain, B. P. Unfolded protein response signaling in liver disorders: a 2023 updated review. *Int. J. Mo.l Sci.*10.3390/ijms241814066 (2023). 10.3390/ijms241814066 PMC10531763 37762367

[34] Yang, Y., Chauhan, B. K., Cveklova, K. & Cvekl, A. Transcriptional regulation of mouse alphaB- and gammaF-crystallin genes in lens: opposite promoter-specific interactions between Pax6 and large Maf transcription factors. *J. Mol. Biol.* **344**, 351–368 (2004). 15522290 10.1016/j.jmb.2004.07.102

[35] He, J. et al. Hspb1 protects against severe acute pancreatitis by attenuating apoptosis and ferroptosis via interacting with Anxa2 to restore the antioxidative activity of Prdx1. *Int. J. Biol. Sci.* **20**, 1707–1728. 10.7150/ijbs.84494 (2024). 38481805 10.7150/ijbs.84494 PMC10929186

[36] Djamali, A., Reese, S., Oberley, T., Hullett, D. & Becker, B. Heat shock protein 27 in chronic allograft nephropathy: a local stress response. *Transplantation* **79**, 1645–1657. 10.1097/01.tp.0000164319.83159.a7 (2005). 15973165 10.1097/01.tp.0000164319.83159.a7

[37] Nahomi, R. B., Palmer, A., Green, K. M., Fort, P. E. & Nagaraj, R. H. Pro-inflammatory cytokines downregulate Hsp27 and cause apoptosis of human retinal capillary endothelial cells. *Biochim. Biophys. Acta.* **164–174**, 2014. 10.1016/j.bbadis.2013.11.011 (1842). 10.1016/j.bbadis.2013.11.011 PMC3905326 24252613

[38] Walter, F., O’Brien, A., Concannon, C. G., Dussmann, H. & Prehn, J. H. M. ER stress signaling has an activating transcription factor 6alpha (ATF6)-dependent “off-switch”. *J. Biol. Chem.* **293**, 18270–18284. 10.1074/jbc.RA118.002121 (2018). 30287689 10.1074/jbc.RA118.002121 PMC6254332

[39] Ramaekers, F. C., Benedetti, E. L., Dunia, I., Vorstenbosch, P. & Bloemendal, H. Polyribosomes associated with microfilaments in cultured lens cells. *Biochim. Biophys. Acta.* **740**, 441–448 (1983). 6309238 10.1016/0167-4781(83)90093-3

[40] Oyadomari, S. & Mori, M. Roles of CHOP/GADD153 in endoplasmic reticulum stress. *Cell Death Differ.* **11**, 381–389. 10.1038/sj.cdd.4401373 (2004). 14685163 10.1038/sj.cdd.4401373

[41] Germain, D. Sirtuins and the estrogen receptor as regulators of the mammalian mitochondrial UPR in cancer and aging. *Adv. Cancer Res.* **130**, 211–256. 10.1016/bs.acr.2016.01.004 (2016). 27037754 10.1016/bs.acr.2016.01.004

[42] Xu, S. et al. Dual roles of UPR(er) and UPR(mt) in neurodegenerative diseases. *J. Mol. Med. (Berl)* **101**, 1499–1512. 10.1007/s00109-023-02382-9 (2023). 37817014 10.1007/s00109-023-02382-9

[43] Scher, M. B., Vaquero, A. & Reinberg, D. SirT3 is a nuclear NAD+-dependent histone deacetylase that translocates to the mitochondria upon cellular stress. *Genes Dev* **21**, 920–928. 10.1101/gad.1527307 (2007). 17437997 10.1101/gad.1527307 PMC1847710

[44] Hu, S. et al. Nrf1 is an indispensable redox-determining factor for mitochondrial homeostasis by integrating multi-hierarchical regulatory networks. *Redox Biol.* **57**, 102470. 10.1016/j.redox.2022.102470 (2022). 36174386 10.1016/j.redox.2022.102470 PMC9520269

[45] Bourougaa, K. et al. Endoplasmic reticulum stress induces G2 cell-cycle arrest via mRNA translation of the p53 isoform p53/47. *Mol. Cell* **38**, 78–88. 10.1016/j.molcel.2010.01.041 (2010). 20385091 10.1016/j.molcel.2010.01.041

[46] Lopez, I. et al. p53-mediated suppression of BiP triggers BIK-induced apoptosis during prolonged endoplasmic reticulum stress. *Cell Death Differentiation* **24**, 1717–1729. 10.1038/cdd.2017.96 (2017). 28622297 10.1038/cdd.2017.96 PMC5596431

[47] Phang, B. H. et al. Amino-terminal p53 mutations lead to expression of apoptosis proficient p47 and prognosticate better survival, but predispose to tumorigenesis. *Proc. Natl. Acad. Sci. U. S. A.* **112**, E6349-6358. 10.1073/pnas.1510043112 (2015). 26578795 10.1073/pnas.1510043112 PMC4655557

[48] Yin, Y., Stephen, C. W., Luciani, M. G. & Fahraeus, R. p53 Stability and activity is regulated by Mdm2-mediated induction of alternative p53 translation products. *Nat. Cell Biol.* **4**, 462–467. 10.1038/ncb801 (2002). 12032546 10.1038/ncb801

[49] Xie, W. et al. Chaperone-mediated autophagy prevents apoptosis by degrading BBC3/PUMA. *Autophagy* **11**, 1623–1635. 10.1080/15548627.2015.1075688 (2015). 26212789 10.1080/15548627.2015.1075688 PMC4590652

[50] Wang, J. et al. Puma, noxa, p53, and p63 differentially mediate stress pathway induced apoptosis. *Cell Death Dis* **12**, 659. 10.1038/s41419-021-03902-6 (2021). 34193827 10.1038/s41419-021-03902-6 PMC8245518

[51] Toruno, C., Carbonneau, S., Stewart, R. A. & Jette, C. Interdependence of Bad and Puma during ionizing-radiation-induced apoptosis. *PLoS One* **9**, e88151. 10.1371/journal.pone.0088151 (2014). 24516599 10.1371/journal.pone.0088151 PMC3916415

[52] Verhagen, A. M., Coulson, E. J. & Vaux, D. L. Inhibitor of apoptosis proteins and their relatives: IAPs and other BIRPs. *Genome Biol.* **2**, REVIEWs3009. 10.1186/gb-2001-2-7-reviews3009 (2001). 11516343 10.1186/gb-2001-2-7-reviews3009 PMC139420

[53] Gortat, A. et al. Apaf1 inhibition promotes cell recovery from apoptosis. *Protein Cell* **6**, 833–843. 10.1007/s13238-015-0200-2 (2015). 26361785 10.1007/s13238-015-0200-2 PMC4624680

[54] Wawrousek, E. F., Chepelinsky, A. B., McDermott, J. B. & Piatigorsky, J. Regulation of the murine alpha A-crystallin promoter in transgenic mice. *Dev. Biol.* **137**, 68–76 (1990). 2295367 10.1016/0012-1606(90)90008-7

[55] Kramer, A., Green, J., Pollard, J. Jr. & Tugendreich, S. Causal analysis approaches in ingenuity pathway analysis. *Bioinformatics* **30**, 523–530. 10.1093/bioinformatics/btt703 (2014). 24336805 10.1093/bioinformatics/btt703 PMC3928520

[56] Ong, G. & Logue, S. E. Unfolding the interactions between endoplasmic reticulum stress and oxidative stress. *Antioxidants (Basel)*10.3390/antiox12050981 (2023). 37237847 10.3390/antiox12050981 PMC10215201

[57] Gebert, M., Slawski, J., Kalinowski, L., Collawn, J. F. & Bartoszewski, R. The unfolded protein response: a double-edged sword for brain health. *Antioxidants (Basel)*10.3390/antiox12081648 (2023). 37627643 10.3390/antiox12081648 PMC10451475

