# Supplementary material for: The c.119-123dup5bp mutation in human γC-crystallin destabilizes the protein and activates the unfolded protein response to cause highly variable cataracts
Source: Sci Rep. 2025 Feb 24;15:6542. doi: 10.1038/s41598-025-90977-2 (PMC11850903; doi:10.1038/s41598-025-90977-2)
Supplement: Supplementary file 1 — Supplementary Information. [file 41598_2025_90977_MOESM1_ESM.docx]

Supplemental Figure Legends

Fig. S1. Alternate splicing of Tp53 in MUT-Severe lenses. A. Overview of RNA-Seq results showing the individual samples (WT: WC 33_trim, WC65_trim, and WC67_trim); MUT-Clear: MC37_trim, MC39_trim, MC42_trim; MUT-Severe: MS55_trim, MS56_trim, MS58_trim) above and the averaged WT (WTC), MUT-Clear (MutC) and MUT-Severe (MutS) depths of sequences below. B. Detail of the 5’ end of exon 1 showing the depths of the individual samples above and the averaged values below. While there is slightly more depth in the MUT-Severe (MutS) lenses this is not changed from the main body of exon 1. C. Detail of the 5’ end of the final exon showing increased expression of the Trp53-202 and Trp53-206 isoforms in the MUT-Severe (MutS) lenses. Trp53-205 is a noncoding RNA expressed in multiple tissues including the lens.

Figure S2. Relative Expression Levels of Chaperones in Mut-Severe (MS) vs. Mut-Clear (MC) vs. WT Lenses. Expression values are normalized to 1 relative to that in WT lenses, where the rpk values are, Hspb1: 14589.6, Cryaa: 3521730, Cryab: 611246.33. *: p = 0.002, ^: p = 0.005

Supplemental Table legends

Table S1. List of genes identified in the RNASeq analysis. The Ensemble and gene symbols are followed by the average expression values for WT, MT-Clear (MC), and MT-Severe (MS) lenses in rpkm, then the ratios of the three groups and their corresponding p values as estimated by a Student’s T test.

Table S2. List of IPA canonical pathways and their corresponding activation in wild type (WT), MT-Clear (MC), and severe mutant (MS) lenses as shown by the corresponding Z scores as estimated by IPA pathway analysis.

Table S3. List of primers used for genotyping and quantitative real time PCR.
